# Clinical characteristics associated with hepatic steatosis on ultrasonography in patients with elevated alanine aminotransferase

**DOI:** 10.1590/S1516-31802010000600006

**Published:** 2010-12-02

**Authors:** Janaína Luz Narciso-Schiavon, Leonardo de Lucca Schiavon, Roberto José de Carvalho, Débora Yumi Hayashida, Jenny Hue Jiuan Wang, Tatiana Santana Souza, Christini Takemi Emori, Maria Lucia Gomes Ferraz, Antonio Eduardo Benedito Silva

**Affiliations:** I MD, PhD. Associate researcher, Hepatitis Section, Division of Gastroenterology, Universidade Federal de São Paulo (Unifesp), São Paulo, Brazil.; II Medical student, Hepatitis Section, Division of Gastroenterology, Universidade Federal de São Paulo (Unifesp), São Paulo, Brazil.; III MD. Postgraduate student, Hepatitis Section, Division of Gastroenterology, Universidade Federal de São Paulo (Unifesp), São Paulo, Brazil.; IV MD, PhD. Associate professor, Hepatitis Section, Division of Gastroenterology, Universidade Federal de São Paulo (Unifesp), São Paulo, Brazil.

**Keywords:** Fatty liver, Alanine transaminase, Ultrasonography, Diabetes mellitus, Body mass index, Fígado gorduroso, Alanina transaminase, Ultrassonografia, Diabetes mellitus, Índice de massa corporal

## Abstract

**CONTEXT AND OBJECTIVE:**

The main causes of hepatic steatosis (HS) are alcoholic liver disease and nonalcoholic fatty liver disease (NAFLD). Although liver biopsy is the gold standard for NAFLD diagnosis, the finding of abnormal aminotransferases in abstinent individuals, without known liver disease, suggests the diagnosis of NAFLD in 80-90% of the cases. Identification of clinical factors associated with HS on abdominal ultrasound may enable diagnoses of fatty liver non-invasively and cost-effectively. The aim here was to identify clinical variables associated with HS in individuals with elevated alanine aminotransferase (ALT) levels.

**DESIGN AND SETTING:**

Cross-sectional study in a single tertiary care center.

**METHODS:**

Individuals with elevated ALT, serologically negative for hepatitis B and C, were evaluated by reviewing medical files. Patients who did not undergo abdominal ultrasonography were excluded.

**RESULTS:**

Among 94 individuals included, 40% presented HS on ultrasonography. Compared with individuals without HS, those with fatty liver were older (P = 0.043), with higher body mass index (BMI) (P = 0.003), diabetes prevalence (P = 0.024), fasting glucose levels (P = 0.001) and triglycerides (P = 0.003). Multivariate analysis showed that BMI (odds ratio, OR = 1.186; 95% confidence interval, CI: 1.049-1.341; P = 0.006) and diabetes mellitus (OR = 12.721; 95% CI: 1.380-117.247; P = 0.025) were independently associated with HS.

**CONCLUSIONS:**

Simple clinical findings such as history of diabetes and high BMI may predict the presence of HS on ultrasonography in individuals with elevated ALT and negative serological tests for hepatitis.

## RESUMO

**CONTEXTO E OBJETIVO:**

Doença hepática alcoólica e doença hepática esteatótica não alcoólica (DHENA) são as principais causas de esteatose hepática (EH). Apesar de a biópsia hepática ser o método de escolha para diagnóstico DHENA, o achado de aminotransferases elevadas em indivíduos abstêmios, sem doença hepática conhecida, sugere o diagnóstico de DHENA em 80-90% dos casos. A identificação de variáveis clínicas associadas à EH na ultrassonografia abdominal pode permitir o diagnóstico de DHENA de forma não invasiva e custo-efetiva. O objetivo foi identificar variáveis clínicas associadas à EH em indivíduos com níveis elevados de alanina aminotransferase (ALT).

**TIPO DE ESTUDO E LOCAL:**

Estudo transversal em um único centro de atendimento terciário.

**MÉTODOS:**

Indivíduos com ALT elevada e sorologias negativas para os vírus de hepatite B e C foram avaliados por meio de revisão de prontuários. Os pacientes não submetidos à ultrassonografia foram excluídos.

**RESULTADOS:**

Foram incluídos 94 indivíduos, 40% deles com EH à ultrassonografia. Quando comparados aos indivíduos sem EH, aqueles com EH apresentaram maior prevalência de diabetes (P = 0,024), maiores idade (P = 0,043) e índice de massa corpórea (IMC) (P = 0,003), glicemia de jejum mais elevada (P = 0,001) e triglicerídeos mais elevados (P = 0,003). A análise multivariada evidenciou que o IMC (*odds ratio*, OR = 1,186, 95% intervalo de confiança, IC 1,049-1,341, P = 0,006) e o diabetes mellitus (OR = 12,721, 95% IC 1,380-117,247, P = 0,025) foram associadas independentemente à EH.

**CONCLUSÕES:**

Achados clínicos simples como história de diabetes e o IMC elevado podem predizer a presença de EH à ultrassonografia de indivíduos com ALT elevada e sorologias negativas para hepatite.

## INTRODUCTION

Hepatic steatosis is a generic term that refers to the accumulation of triglycerides in the cytoplasm of hepatocytes. Fatty liver is usually diagnosed in individuals without clinical evidence of liver disease, after fortuitous identification of elevated serum aminotransferase levels. It is an important pathological condition because of its high prevalence, affecting about 25 to 35% of the United States adult population.^1^ The main conditions associated with the presence of fatty liver are alcoholic liver disease and nonalcoholic fatty liver disease (NAFLD).^2^ Fatty liver can also occur due to various other etiological factors, including the use of medications (such as tamoxifen and methotrexate) and toxins (such as carbon tetrachloride and arsenic) and the presence of chronic viral hepatitis B and C (most commonly in hepatitis C infection) and other metabolic diseases (such as hemochromatosis and Wilson's disease).^3^

The evolution of liver disease is variable, according to the cause of steatosis. In alcoholic liver disease, the stages are similar to those of NAFLD and progress from steatosis to steatohepatitis with varying degrees of fibrosis, which can lead to cirrhosis. In viral hepatitis, liver fibrosis progression to cirrhosis is due to the presence of chronic active hepatitis.^4^ Even if the evolution of NAFLD depends on the causative factor, the early stages may be reversible if early intervention is taken to remove the offending factor. Individuals with advanced fibrosis exhibit an increased risk of developing hepatocellular carcinoma, although this risk is less common in NAFLD than is seen in cirrhosis due to alcohol or the hepatitis C virus.^5^

NAFLD is the most common cause of hepatic steatosis, along with elevated aminotransferase levels, and is present in 2.8 to 5.5 percent of the population.^2^ Suspected NAFLD is among the leading causes of outpatient consultations with gastroenterologists and hepatologists.^2,6^ Although some patients are found to present painful hepatomegaly, the vast majority are asymptomatic, and liver disease is identified incidentally from routine laboratory tests or imaging. Its initial assessment consists of ruling out other causes of liver disease and identifying clinical comorbidities, particularly metabolic disorders.^7^

The risk factors for NAFLD include obesity, diabetes mellitus, insulin resistance, dyslipidemia and systemic hypertension.^6,8,9^ However, not all patients with metabolic syndrome develop hepatic steatosis. Laboratory tests frequently show changes in liver damage markers, particularly alanine aminotransferase (ALT), aspartate aminotransferase (AST) and gamma-glutamyltransferase (GGT).^6,8-9^ In NAFLD, the ALT level is not correlated with the degree of histological activity and may be normal, even in the presence of advanced disease.^9^

Although liver biopsy is considered to be the gold standard method for diagnosing and staging NAFLD, the finding of abnormal liver enzyme levels in individuals who do not abuse alcohol and do not have any known liver disease or risk factors suggests the diagnosis of NAFLD in 80-90% of the cases.^8^ Identification of clinical and laboratory features associated with steatosis on abdominal ultrasound may make it possible to diagnose fatty liver in a non-invasive and cost-effective manner.

## OBJECTIVE

The objective of this study was to identify the clinical and laboratory variables associated with steatosis on ultrasound examinations, among blood donors with elevated ALT and negative serological tests for hepatitis B and C viruses.

## MATERIALS AND METHODS

### Patients

This cross sectional study was carried out in a single tertiary care center (Hepatitis Section, Hospital São Paulo) and included donors who had been referred by the blood bank because they presented elevated ALT levels but were serologically negative for hepatitis B and C (hepatitis B surface antigen [HBsAg], antibody to hepatitis B core antigen [anti-HBc] and anti-hepatitis C virus antibody [anti-HCV]), between September 1997 and August 2006. Patients who did not undergo abdominal ultrasonography were excluded.

The study protocol conformed to the ethical guidelines of the 1975 Helsinki Declaration and was approved by our institutional review board.

### Methods

Demographic, laboratory, ultrasound and other clinical variables were reviewed and extracted from standardized medical records. Only clinical data, laboratory tests and ultrasonography obtained within three months from the first medical consultation were used for this study. The patients included were analyzed for the following clinical and epidemiological characteristics: gender, age, body mass index (BMI), history of diabetes mellitus, history of dyslipidemia, alcohol abuse, occupational activity (healthcare field or other activity), risky sexual behavior (defined by identifying promiscuity [three or more partners within six months], previous sexually transmitted disease or homosexuality), and exposure to parenteral risk (characterized by parenteral use of drugs with sharing of injection equipment [intravenous drugs or illegal energy substances] or transfusion of blood components). History of diabetes mellitus was defined by previously diagnosed fasting glucose ≥ 126 mg/dl or glucose ≥ 200 mg/dl at any time, associated with symptoms or detected in an oral glucose tolerance test. History of dyslipidemia was defined as a prior diagnosis of hypertriglyceridemia (triglycerides ≥ 150 mg/dl) and/or hypercholesterolemia (total cholesterol ≥ 200 mg/dl) and/or HDL < 50 mg/dl in women and < 40 mg/dl in men. Alcohol abuse was defined as reporting of alcohol consumption exceeding 20 g per day for women and 30 g per day for men one year prior to enrollment. Ultrasound data, regarding the presence or absence of hepatic steatosis, was obtained from the medical records.

With regard to the biochemical variables analyzed, ALT, AST, alkaline phosphatase (ALP) and GGT were expressed in the form of an index calculated as the ratio between the values obtained and the upper limit of normality (ULN). Thus, a test result was considered high when the resulting value was equal to or more than one ULN. The other parameters were expressed as absolute values.

### Statistical analysis

Continuous variables were compared using Student's t test, or the Mann-Whitney U test when appropriate. Categorical variables were compared using Pearson's c^2^ test or Fisher's exact test. P-values of less than 0.05 were considered statistically significant. Bivariate analysis and regression analysis were used to identify variables associated with the presence of liver steatosis on ultrasound. All tests were two-tailed and were performed using the Statistical Package for the Social Sciences (SPSS) software, version 15.0 (SPSS, Chicago, Illinois, USA).

## RESULTS

### Patient characteristics

**Figure 1.**
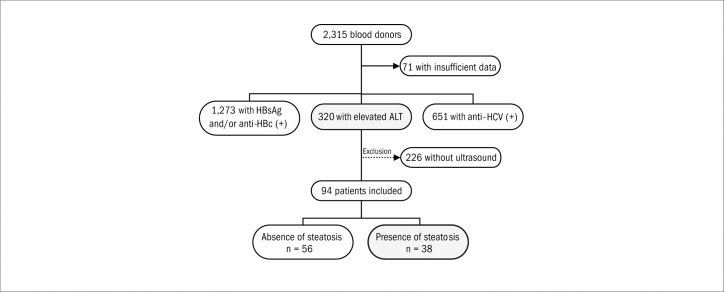
Distribution of potential candidates for participation in the study, candidates excluded and reasons for exclusion.

During the study period, which covered nine years, 2,315 blood donors were referred from blood banks to the Hepatitis Division of Universidade Federal de São Paulo (Unifesp), Brazil, with abnormal liver test results. Among the subjects studied, 71 had insufficient data in the records. With regard to the reason for referral, 320 donors (14.3%) were attended because of high levels of ALT; 1,273 (56.7%) because of HBsAG and/or anti-HBc positive or inconclusive tests; and 651 (29.0%) because of reactive or inconclusive anti-HCV results. Among the 320 subjects with elevated ALT levels and negative serological test results for viral hepatitis considered for inclusion in the study, 226 were excluded because they had not undergone abdominal ultrasound. The patient distribution is shown in Figure 1.

The mean age was 37.0 ± 9.5 years, the mean BMI was 29.6 ± 4.6 kg/m^2^ and male predominance was observed (88%). Ten percent of the patients had previously been diagnosed with diabetes, 54% had dyslipidemia and 26% had a history of alcohol abuse. The characteristics of the 94 individuals included in the study are shown in Table 1. Given the high number of patients excluded, a comparative analysis was performed between them and the individuals included in the study (those who underwent ultrasonography) (Table 1). Overall, demographic and clinical characteristics were similar between the included and excluded individuals. The patients included were older than the patients excluded (37.0 ± 9.5 versus 34.1 ± 8.9; P = 0.010).

**Table 1.** Comparative analysis on 94 patients included in the study and 226 individuals excluded, regarding their clinical, epidemiological and biochemical characteristics

Characteristics	Excluded from the study n = 226	Included in the study n = 94	P*
Male, n (%)	206 (91.2)	83 (88.3)	0.432
Age (years)†	34.1 ± 8.9 (33.0)	37.0 ± 9.5 (35.0)	**0.010**
BMI (kg/m²)†	28.9 ± 4.4 (28.6)	29.6 ± 4.6 (28.3)	0.206
Diabetes, n (%)	11 (5.4)	9 (10.1)	0.141
Dyslipidemia, n (%)	92 (50.8)	48 (53.9)	0.631
Alcohol abuse, n (%)	84 (37.7)	24 (26.4)	0.056
Health professionals, n (%)	5 (2.2)	1 (1.1)	0.675
Risky sexual behavior, n (%)	53 (23.8)	30 (33.0)	0.093
Exposure to parental risk, n (%)	6 (2.7)	3 (3.3)	0.724
AST (xLSN)†	1.4 ± 2.7 (1.0)	1.1 ± 0.8 (0.9)	0.150
ALT (xLSN)†	1.9 ± 1.6 (1.6)	1.6 ± 0.6 (1.5)	0.237
DB (mg/dL)†	0.3 ± 0.3 (0.3)	0.3 ± 0.1 (0.3)	0.372
ALP (xLSN)†	0.7 ± 0.3 (0.7)	0.7 ± 0.2 (0.7)	0.785
GGT (xLSN)†	2.1 ± 1.8 (1.4)	2.1 ± 1.7 (1.5)	0.483
Prothrombin activity (%)†	92.8 ± 9.5 (97.5)	92.6 ± 9.7 (100.0)	0.962
Albumin (g/dl)†	4.4 ± 0.4 (4.4)	4.5 ± 0.5 (4.5)	0.415
Platelets (/mm³)†	231,200 ± 54,073 (222,000)	230,340 ± 50,408 (230,000)	0.920
Fasting glucose (g/dl)	99.4 ± 34.7 (91.0)	106.9 ± 42.0 (96.5)	0.082
Total cholesterol (mg/dl)	215.2 ± 53.4 (210.0)	202.0 ± 40.2 (198.5)	0.053
HDL cholesterol (mg/dl)	43.9 ± 12.2 (43.0)	44.1 ± 10.8 (42.5)	0.430
LDL cholesterol (mg/dl)	130.9 ± 41.7 (136.0)	123.1 ± 34.9 (124.0)	0.174
Triglycerides	221.9 ± 254.5 (160.0)	170.8 ± 100.27 (148.0)	0.367

BMI = body mass index; AST = aspartate aminotransferase; ALT = alanine aminotransferase; DB = direct bilirubin; ALP = alkaline phosphatase; GGT = gamma glutamyltransferase;

* Student's *t* test, Mann-Whitney test, χ^2^ test or Fisher's exact test, when appropriate for group comparisons;

† Mean ± standard deviation and median.

### Factors associated with liver steatosis

Comparison of individuals with hepatic steatosis and those who did not have steatosis on ultrasound showed that the individuals with steatosis were older (39.3 ± 10.0 versus 35.3 ± 8.8 years; P = 0.043) and had higher BMI (31.6 ± 5.3 versus 28.4 ± 3.6 kg/m^2^; P = 0.003) and higher prevalence of diabetes (20.6% versus 3.6%; P = 0.024). The distribution of the clinical and epidemiological characteristics of the 94 patients according to the presence of steatosis on ultrasonography is shown in Table 2. Patients with steatosis on ultrasonography showed higher mean fasting glucose levels (118.4 ± 47.7 versus 98.7 ± 35.8 mg/dl; P = 0.001) and higher mean triglyceride levels (203.9 ± 104.9 versus 147.6 ± 90.9 mg/dl; P = 0.003). Biochemical variables according to the presence of steatosis at ultrasonography are shown in Table 3.

**Table 2.** Distribution of clinical and epidemiological characteristics among 94 blood donors with elevated ALT, according to the presence of hepatic steatosis on ultrasonography

Characteristics	Ultrasonography		P*
	Absence of steatosis n = 56 (60%)	Presence of steatosis n = 38 (40%)	
Male, n (%)	51 (91.1)	32 (84.2)	0.342
Age (years)^†^	35.3 ± 8.8 (35.0)	39.3 ± 10.0 (37.5)	**0.043**
BMI (kg/m²)^†^	28.4 ± 3.6 (27.7)	31.6 ± 5.3 (30.7)	**0.003**
Diabetes, n (%)	3 (3.6)	7 (20.6)	**0.024**
Dyslipidemia, n (%)	27 (50.0)	21 (60.0)	0.355
Alcohol abuse, n (%)	17 (32.1)	7 (18.4)	0.145
Health professionals, n (%)	0 (0.0)	1 (2.6)	0.409
Risky sexual behavior, n (%)	18 (34.0)	12 (31.6)	0.811
Exposure to parental risk, n (%)	1 (1.9)	2 (5.3)	0.567

ALT = alanine aminotransferase; BMI = body mass index;

* Mean ± standard deviation and median;

‡ Student's *t* test, Mann-Whitney test, χ^2^ test or Fisher's exact test, as appropriate for group comparisons.

**Table 3.** Distribution of biochemical variables among 94 blood donors with elevated ALT, according to the presence of hepatic steatosis on ultrasonography

Biochemical variables	Ultrasonography		P*
	Absence of steatosis n = 56	Presence of steatosis n = 38	
AST (xLSN)†	1.0 ± 0.7 (0.9)	1.6 ± 0.9 (0.9)	0.500
ALT (xLSN)†	1.6 ± 0.6 (1.5)	1.6 ± 0.6 (1.5)	0.695
DB (mg/dl)†	0.3 ± 0.1 (0.3)	0.3 ± 0.1 (0.3)	0.880
ALP (xLSN)†	0.7 ± 0.2 (0.6)	0.7 ± 0.3 (0.7)	0.103
GGT (xLSN)†	2.3 ± 1.8 (1.8)	1.9 ± 1.6 (1.3)	0.369
Prothrombin activity (%)†	94.0 ± 8.7 (100.0)	90.8 ± 11.0 (97.4)	0.270
Albumin (g/dl)†	4.5 ± 0.5 (4.5)	4.4 ± 0.6 (4.5)	0.908
Platelets (/mm³)†	221,450 ± 50,890 (221,500)	244,420 ± 47,283 (251,000)	0.080
Fasting glucose (g/dl)	98.7 ± 35.8 (91.5)	118.4 ± 47.7 (105.0)	**0.001**
Total cholesterol (mg/dl)	200.7 ± 43.3 (196.0)	203.9 ± 35.7 (204.0)	0.723
HDL cholesterol (mg/dl)	44.8 ± 12.0 (42.5)	43.1 ± 8.8 (42.5)	0.785
LDL cholesterol (mg/dl)	124.2 ± 38.2 (124.5)	121.5 ± 29.9 (118.0)	0.749
Triglycerides (mg/dl)	147.6 ± 90.9 (113.0)	203.9 ± 104.9 (174.0)	**0.003**

xLSN = times the upper limit of normality; AST = aspartate aminotransferase; ALT = alanine aminotransferase; DB = direct bilirubin; ALP = alkaline phosphatase; GGT = gamma glutamyltransferase;

* Student's t test or Mann-Whitney test, as appropriate for group comparisons.

† Mean ± standard deviation and median.

Multivariate analysis showed that BMI (odds ratio, OR = 1.186; 95% confidence interval, CI: 1.049 to 1.341; P = 0.006) and history of diabetes mellitus (OR = 12.721; 95% CI: 1.380 to 117.247; P = 0.025) were independently associated with the presence of steatosis on ultrasonography.

## DISCUSSION

Investigation of factors associated with steatosis has been highlighted in the medical literature over recent years. This is because of its high prevalence: it is the most common cause of chronic liver disease among adults and children in the United States.^10^

The prevalence of fatty liver disease depends on the population studied and the method used for diagnosis. In the general population, the prevalence of fatty liver disease is 16 to 29% on ultrasonography,^11^ 31 to 34% on magnetic resonance imaging (MRI)^1,12^ and 15 to 39% on liver biopsy.^13^

Liver biopsy remains the gold standard method for diagnosing and staging fatty liver, since it enables fat quantification and identification of steatohepatitis and different degrees of fibrosis.^14^ However, it is an invasive procedure, and the morbidity rates are certainly not negligible. Among the potentially serious complications, internal bleeding, bladder perforation, peritonitis, hematoma and infection can be mentioned. In fact, about 3% of the patients require hospitalization after undergoing liver biopsy.^15^ Pain following the procedure and high cost are common causes of dissatisfaction. Furthermore, studies have shown that histological evaluations may be complicated by the possibility of sampling error and by considerable interobserver and intraobserver variability. Liver biopsies allow histological analysis on 1/50,000 of the liver.^16^ The distribution of lipid deposits in fatty liver cases may be heterogeneous, and therefore, a single liver biopsy may not adequately represent the disease throughout the liver.^17^ Hence, special attention has been given to noninvasive methods for identifying and quantifying hepatic steatosis. The conventional methods include abdominal ultrasonography, computed tomography (CT) and MRI .^4^

Ultrasonography is the method of choice for the initial assessment of hepatic steatosis, since it presents a number of advantages over other imaging methods: low cost, safety, non-use of intravenous contrast, wide availability and widespread acceptance by patients.^18^ In the presence of hepatic steatosis, hyperechogenicity is observed on ultrasound examination in the liver parenchyma, associated with changes in echo texture, vascular blurring and deep attenuation. This corresponds to steatotic infiltration greater than 30% in both liver lobes, with sensitivity of 60 to 95% and specificity of 77 to 100%.^18^

NAFLD prevalence increases with age, type 2 diabetes mellitus, obesity and hypertriglyceridemia.^6,19^ The association of NAFLD with gender is controversial: earlier studies indicated that NAFLD is more frequent among women, possibly because these studies included individuals referred to gastroenterologists. More recently, in population-based studies, there has not been any such relationship.^19^ Visceral obesity, defined by a high waist-hip ratio, is also considered to be a risk factor for NAFLD. The presence of hyperinsulinemia or insulin resistance and associations with some components of metabolic syndrome suggest that NAFLD may be the hepatic manifestation of metabolic syndrome.^20^ Most studies have evaluated the risk factors for NAFLD using liver biopsy, but few studies have evaluated the risk factors for the presence of fatty liver on ultrasonography.

This study demonstrated that BMI and history of diabetes mellitus were independently associated with steatosis on abdominal ultrasound examination. Similar results were found by Ryan et al.,^21^ who assessed the presence of fatty liver on ultrasonography among HIV-positive individuals. Hepatic steatosis is present in two thirds of the obese population, regardless of the presence of diabetes,^22^ and in more than 90% of morbidly obese individuals.^23^ Not only is obesity a predisposing factor for the emergence of type 2 diabetes and metabolic syndrome itself, but also its association with diabetes may represent an additional risk for the development of NAFLD. In a study evaluating obese patients with diabetes, 100% had steatosis, 50% had steatohepatitis and 19% had cirrhosis.^23^

This study has some limitations. Firstly, the retrospective data collection and the large number of excluded patients may have led to selection bias. However, the data were collected from standardized medical files by a single researcher and the characteristics of the individuals excluded from the study were generally similar to those of the individuals included. Secondly, the strict inclusion of subjects with elevated ALT could represent a limitation, because it is known that individuals with normal ALT may have hepatic steatosis. However, ALT is used as part of the laboratory tests requested for checkups, and because abnormal ALT levels suggest that liver disease is present, it is a frequent cause of referral to specialists. For this reason, the patients included in this study represent a valid population for evaluation. Finally, the use of ultrasound for diagnosing fatty liver is a less sensitive and specific method than liver biopsy. Ultrasound is also an examiner-dependent method, and it was not performed by a single examiner. However, as mentioned earlier, ultrasonography has an excellent cost-effectiveness ratio and it is widely available and easy to perform. For these reasons, it is the preferred test for use in the initial evaluation on patients with ALT elevation.^24,25^ Furthermore, the fact that the ultrasound data was obtained from the medical records may represent an advantage, since this reflects day-to-day clinical practice.

## CONCLUSIONS

In conclusion, hepatic steatosis is frequently found in patients referred because of elevated ALT and negative serological tests for viral hepatitis. Simple clinical findings such as a history of diabetes mellitus and high BMI may predict the presence of fatty liver on ultrasonography among these patients.

## References

[1] Browning J, Szczepaniak LS, Dobbins R (2004). Prevalence of hepatic steatosis in an urban population in the United States: impact of ethnicity. Hepatology.

[2] Clark JM, Diehl AM (2003). Defining nonalcoholic fatty liver disease: implications for epidemiologic studies. Gastroenterology.

[3] Hamer OW, Aguirre DA, Casola G (2006). Fatty liver: imaging patterns and pitfalls. Radiographics.

[4] El-Zayadi AR (2008). Hepatic steatosis: a benign disease or a silent killer. World J Gastroenterol.

[5] Hui JM, Kench JG, Chitturi S (2003). Long-term outcomes of cirrhosis in nonalcoholic steatohepatitis compared with hepatitis C. Hepatology.

[6] Angulo P (2002). Nonalcoholic fatty liver disease. N Engl J Med.

[7] Vuppalanchi R, Chalasani N (2009). Nonalcoholic fatty liver disease and nonalcoholic steatohepatitis: Selected practical issues in their evaluation and management. Hepatology.

[8] Clark JM, Brancati FL, Diehl AM (2002). Nonalcoholic fatty liver disease. Gastroenterology.

[9] Sanyal AJ, American Gastroenterological Association (2002). AGA technical review on nonalcoholic fatty liver disease. Gastroenterology.

[10] Marchesini G, Bugianesi E, Forlani G (2003). Nonalcoholic fatty liver, steatohepatitis, and the metabolic syndrome. Hepatology.

[11] Bedogni G, Miglioli L, Masutti F (2005). Prevalence of and risk factors for nonalcoholic fatty liver disease: the Dionysos nutrition and liver study. Hepatology.

[12] Szczepaniak L, Nurenberg P, Leonard D (2005). Magnetic resonance spectroscopy to measure hepatic triglyceride content: prevalence of hepatic steatosis in the general population. Am J Physiol Endocrinol Metab.

[13] Hilden M, Christoffersen P, Juhl E, Dalgaard JB (1977). Liver histology in a ‘normal’ population--examinations of 503 consecutive fatal traffic casualties. Scand J Gastroenterol.

[14] Adams LA, Angulo P, Lindor KD (2005). Nonalcoholic fatty liver disease. CMAJ.

[15] Janes CH, Lindor KD (1993). Outcome of patients hospitalized for complications after outpatient liver biopsy. Ann Intern Med.

[16] Maharaj B, Maharaj RJ, Leary WP (1986). Sampling variability and its influence on the diagnostic yield of percutaneous needle biopsy of the liver. Lancet.

[17] Arun J, Jhala N, Lazenby AJ, Clements R, Abrams GA (2007). Influence of liver biopsy heterogeneity and diagnosis of nonalcoholic steatohepatitis in subjects undergoing gastric bypass. Obes Surg.

[18] Charatcharoenwitthaya P, Lindor KD (2007). Role of radiologic modalities in the management of non-alcoholic steatohepatitis. Clin Liver Dis.

[19] Falck-Ytter Y, Younossi ZM, Marchesini G, McCullough AJ (2001). Clinical features and natural history of nonalcoholic steatosis syndromes. Semin Liver Dis.

[20] Marchesini G, Brizi M, Morselli-Labate AM (1999). Association of nonalcoholic fatty liver disease with insulin resistance. Am J Med.

[21] Ryan P, Blanco F, García-Gascó P (2009). Predictors of severe hepatic steatosis using abdominal ultrasound in HIV-infected patients. HIV Med.

[22] Wanless IR, Lentz JS (1990). Fatty liver hepatitis (steatohepatitis) and obesity: an autopsy study with analysis of risk factors. Hepatology.

[23] Silverman JF, O'Brien KF, Long S (1990). Liver pathology in morbidly obese patients with and without diabetes. Am J Gastroenterol.

[24] Green RM, Flamm S (2002). AGA technical review on the evaluation of liver chemistry tests. Gastroenterology.

[25] Krier M, Ahmed A (2009). The asymptomatic outpatient with abnormal liver function tests. Clin Liver Dis.

